# Right re-redo video-assisted thoracoscopic surgery lower lobectomy with middle lobe preservation for recurrent and metachronous lung adenocarcinoma

**DOI:** 10.1016/j.xjtc.2023.11.016

**Published:** 2023-12-07

**Authors:** Samuele Nicotra, Vincenzo Verzeletti, Giorgio Cannone, Luigi Lione, Federico Rea

**Affiliations:** Thoracic-Surgery Unit, Department of Cardiac, Thoracic, Vascular Sciences, and Public Health, University of Padua, Padua, Italy

**Figure:**
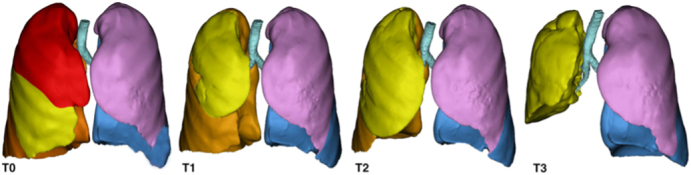
3D reconstructions before (T0) and after the first (T1), the second (T2), and the third (T3) surgery.

**Central Message**

In selected patients affected by recurrent/metachronous lung cancer already surgically resected, middle lobe preservation, instead of pneumonectomy, can represent a valid surgical option.

Recurrent, synchronous, or metachronous lung cancer in a patient already treated with surgery represents a relatively common clinical scenario with a reported incidence ranging from 0.2% to 20%.^1^ In the treatment of these patients, surgeons are still looking for the best way to both radically remove the tumor and preserve the greatest amount of healthy lung tissue.^1^^,^^2^

Herein, we report a unique case of a right a re-redo video-assisted thoracoscopic surgery (VATS) lower lobectomy with middle lobe (ML) preservation in a patient who previously received a right upper lobectomy and a right S10-segmentectomy for a lung adenocarcinoma. We obtained informed consent from the patient for the publication of this work; institutional review board approval was not required.

## Case Presentation

A 68-year-old woman underwent, 6 years earlier, a VATS right upper lobectomy for a lung adenocarcinoma and a redo VATS S10-segmentectomy for a recurrence 3 years after. During follow-up, 5 subcentimeter nodules were detected in the remaining right lower lobe: a transthoracic computed tomography- (CT) guided biopsy was performed, with final diagnosis of lung adenocarcinoma. After a careful preoperative evaluation calculating the ML volume at chest CT scan and lung scintigraphy (right 45% and left [L] 55%), a right lower completion lobectomy with ML preservation was proposed. The patient was even conscious of the possibility of a completion pneumonectomy.

A right triportal re-redo VATS approach was performed. Modest pleuroparenchymal adherences were found and the isolation of hilar structures was demanding because of dense perivascular and peribronchial tissues resulting from the past 2 surgeries (Videos 1 and 2). After right lower lobectomy, the ML confirmed its satisfactory re-expansion without evidence of macroscopic risks of lobar torsion. Therefore, we decided to preserve the ML without a fixation system (Videos 1 and 2). Lymphadenectomy was not performed because it had already been radically performed at the first surgery. The postoperative course was uneventful, the pleural drainage was removed at postoperative day (POD) 4, the air leaks were always <80 mL/min, and self-resolved between POD 3 and 4. The patient was discharged on POD 5. The final pathology examination confirmed the presence of 5 adenocarcinomas; 4 of these were compatible with local recurrence, whereas 1 was classified as metachronous.

The respiratory function tests showed a natural reduction in the respiratory reserve after each surgery, with satisfactory values 3 months after the third operation: forced expiratory volume in 1 second 1.45 L (69%), forcedvital capacity 1.96 L (78%), total lung capacity 3.51 L (72%), residual volume 1.53 L (76%), and diffusing capacity for carbone monoxide 48% (Table 1).

**Table 1:** Volumetric calculations and spirometries at the preoperative (T0) and postoperative time after the first (T1), the second (T2), and the third (T3) surgery

			T0		T1		T2		T3	
ML volume	cm^3^		505.928		380.767		696.103		986.385	
ML expansion	%		–		−24.74		+37.59		+94.96	
ML density	HU		−837.364		−863.581		−852.470		−863.420	
VC	Observed (l)	Obs/Pred (%)	3.40	116.4	2.98	113	2.79	111	1.98	79
FVC	Observed (l)	Obs/Pred (%)	3.40	121.1	2.92	111	2.76	110	1.96	78
FEV1	Observed (l)	Obs/Pred (%)	2.75	116.3	2.51	113	1.97	94	1.45	69
FEV1/FVC	Observed (%)		80.88		85.71		71.55		73.93	
RV	Observed (l)	Obs/Pred (%)	2.06	104	1.95	98	1.75	87	1.53	76
TLC	Observed (l)	Obs/Pred (%)	5.25	102.9	4.93	99	4.55	93	3.51	72
DLCO	Obs/Pred (%)		76		79		73		48	

*T0*, Preoperative surgery; *T1*, postoperative time after the first surgery; *T2*, postoperative time after the second surgery; *T3*, postoperative time after the third surgery; *ML*, middle lobe; *VC*, vital capacity; *Obs/Pred*, observed/prediced; *FVC*, forced vital capacity; *FEV1*, forced espiratory volume; *RV*, residual volume; *TLC*, total lung capacity; *DLCO*, diffusing capacity for carbone monoxide.

**Figure 1:**
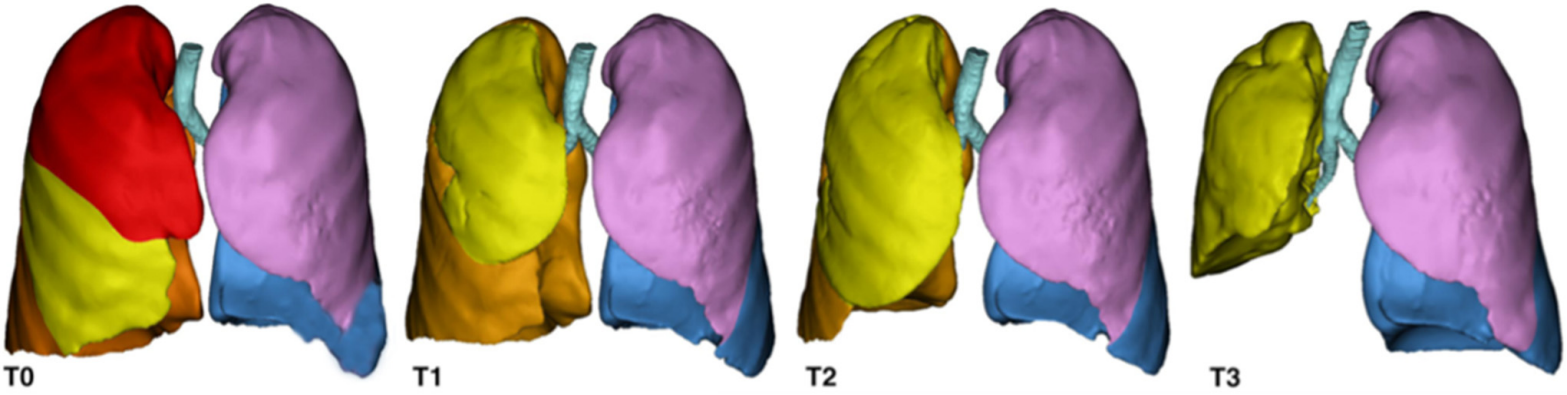
Three-dimensional reconstructions before (*T0*) and after the first (*T1*), the second (*T2*), and the third (*T3*) surgery.

**Figure 2:**
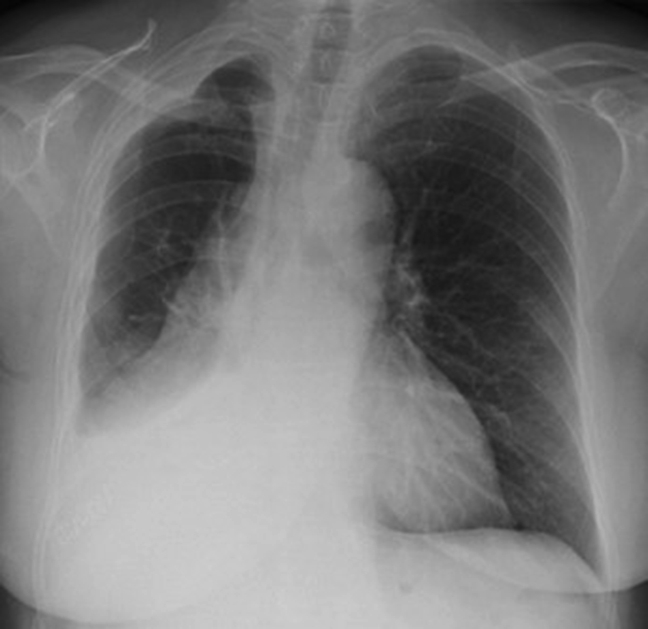
Chest radiographic images after a right re-redo video-assisted thoracoscopic surgery lower lobectomy with middle lobe preservation.

The volumetric calculations made on the preoperative and the postoperative CT scans after the first, the second, and third surgery showed excellent volumetric re-expansion capacity of the ML (see Table 1, Figure 1, and Figure 2). The value at the third surgery (986.385 cm^3^) compared with preoperatively (505.928 cm^3^) was almost twice as much (+94%) (see Table 1 and Figure 1). No signs of mediastinal shift and emphysema were appreciated at the residual ML with a median value of −863.420 HU (see Table 1, Figure 1, and Figure 2). One year after surgery, the patient is healthy, leading an active life without any limitations and with a negative oncology follow-up.

## Discussion

Compromised pulmonary functions by previous surgery, technical surgical difficulties related to redo surgeries, higher rate of postoperative complications, and alternative curative options certainly represent a main issue when faced with a patient with recurrent or metachronous lung cancer.^1^, ^2^, ^3^ However, redo surgery, if the patient is resectable and can tolerate surgery, still represent the standard of care.^1^

With this report, we present an alternative surgical option to avoid a right pneumonectomy, which even nowadays is burdened by high morbidity and mortality.^4^ Conversely, there are some concerns about the real benefit of ML preservation because of its risk of lobar torsion without a real gain in respiratory function. However, some case reports demonstrated that in selected cases, ML might preserve a great amount of lung function, reducing surgery risk and improving postoperative quality of life.^2^^,^^3^

In our case, the idea of attempting to preserve the ML was derived from both its large re-expansion appreciable on preoperative CT scan, its good function assessed on ventiloperfusion scintigraphy, and the excellent intraoperative re-expansion. Particularly, referring to ML re-expansion after each surgery, we curiously observed the ML's volumetric decrease after the first surgery, which could be related to the increased re-expansion of the lower lobe.

It is important to emphasize that this option should be proposed only in selected cases where there is no risk of preserving a nonfunctioning lobe without macroscopic risk of torsion; indeed, if at least 1 of the above conditions is not met, we would recommend pneumonectomy as the treatment of choice.

In our case, the ML was judged to be at low risk of torsion for 2 main reasons: its great expansion observed both preoperatively and postoperatively and because the bronchus intermedius had progressively adapted to the changes in the pleural cavity (ie, major expansion of the ML, the paraphysiologic reduction of the cavity, and the elevation of the diaphragm) related to the 2 previous surgeries.

## Conclusions

In selected patients undergoing reoperation where ML re-expansion is appreciable both on CT scan and after the reinflation at the end of surgery, lung tissue preservation can represent a valid surgical option to preserve a patient's residual respiratory reserve.

## Conflict of Interest Statement

The authors reported no conflicts of interest.

The *Journal* policy requires editors and reviewers to disclose conflicts of interest and to decline handling manuscripts for which they may have a conflict of interest. The editors and reviewers of this article have no conflicts of interest.

## Supplementary Data

Video 1 Part 1. Video available at: https://www.jtcvs.org/article/S2666-2507(23)00467-4/fulltext.

Video 2 Part 2. Video available at: https://www.jtcvs.org/article/S2666-2507(23)00467-4/fulltext.
