# Supplementary figures and images for: Right re-redo video-assisted thoracoscopic surgery lower lobectomy with middle lobe preservation for recurrent and metachronous lung adenocarcinoma
Source: JTCVS Tech. 2023 Dec 7;23:120–2. doi: 10.1016/j.xjtc.2023.11.016 (PMC10859655; doi:10.1016/j.xjtc.2023.11.016)

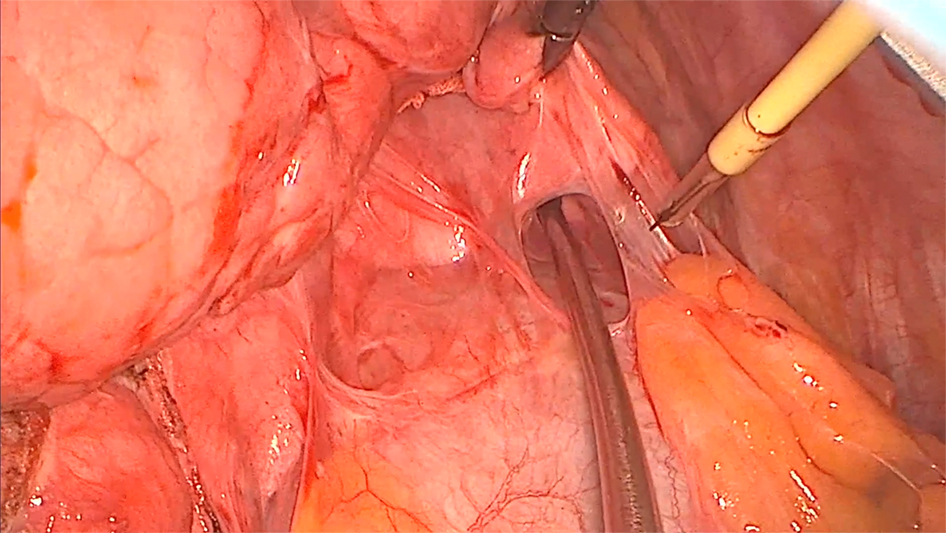

Supplement: Video 1 — Part 1. Video available at: https://www.jtcvs.org/article/S2666-2507(23)00467-4/fulltext. [file fx2.jpg]

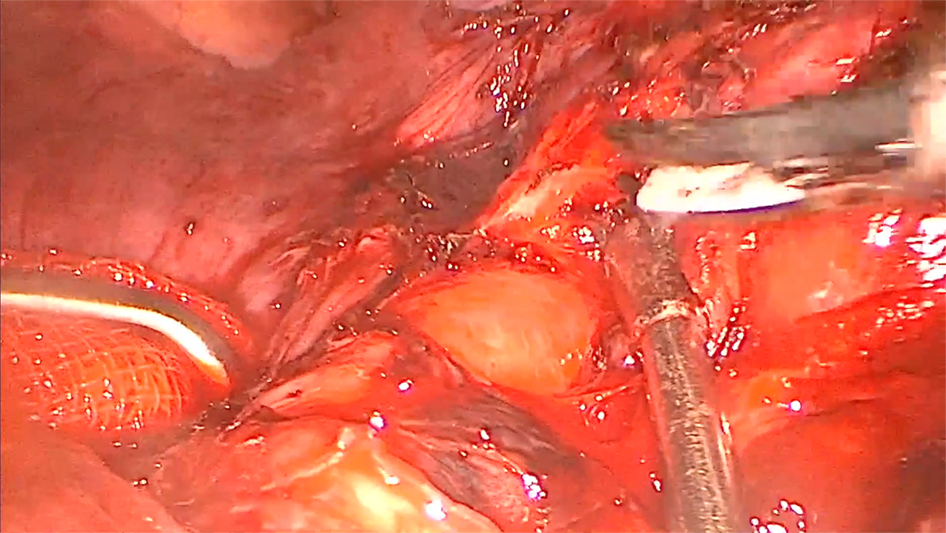

Supplement: Video 2 — Part 2. Video available at: https://www.jtcvs.org/article/S2666-2507(23)00467-4/fulltext. [file fx3.jpg]
